# Can Endovascular Treatment of Fusiform Intracranial Aneurysms Restore the Healthy Hemodynamic Environment?–A Virtual Pilot Study

**DOI:** 10.3389/fneur.2021.771694

**Published:** 2022-01-24

**Authors:** Sylvia Saalfeld, Janneck Stahl, Jana Korte, Laurel Morgan Miller Marsh, Bernhard Preim, Oliver Beuing, Yurii Cherednychenko, Daniel Behme, Philipp Berg

**Affiliations:** ^1^Research Campus STIMULATE, University of Magdeburg, Magdeburg, Germany; ^2^Department of Simulation and Graphics, University of Magdeburg, Magdeburg, Germany; ^3^Department of Fluid Dynamics and Technical Flows, University of Magdeburg, Magdeburg, Germany; ^4^Department of Mechanical Engineering, University of Washington, Seattle, WA, United States; ^5^Department of Radiology, AMEOS Hospital Bernburg, Bernburg, Germany; ^6^Endovascular Centre, Dnipropetrovsk Regional Clinical Hospital named after I.I. Mechnikov, Dnipro, Ukraine; ^7^Department of Neuroradiology, University Hospital Magdeburg, Magdeburg, Germany

**Keywords:** fusiform intracranial aneurysm, hemodynamic simulation, virtual stent deployment, blood flow patterns, endovascular treatment

## Abstract

Numerous studies assess intracranial aneurysm rupture risk based on morphological and hemodynamic parameter analysis in addition to clinical information such as aneurysm localization, age, and sex. However, intracranial aneurysms mostly occur with a saccular shape located either lateral to the parent artery or at a bifurcation. In contrast, fusiform intracranial aneurysms (FIAs), i.e., aneurysms with a non-saccular, dilated form, occur in approximately 3–13% of all cases and therefore have not yet been as thoroughly studied. To improve the understanding of FIA hemodynamics, this pilot study contains morphological analyses and image-based blood flow simulations in three patient-specific cases. For a precise and realistic comparison to the pre-pathological state, each dilation was manually removed and the time-dependent blood flow simulations were repeated. Additionally, a validated fast virtual stenting approach was applied to evaluate the effect of virtual endovascular flow-diverter deployment focusing on relevant hemodynamic quantities. For two of the three patients, post-interventional information was available and included in the analysis. The results of this numerical pilot study indicate that complex flow structures, i.e., helical flow phenomena and the presence of high oscillating flow features, predominantly occur in FIAs with morphologically differing appearances. Due to the investigation of the individual healthy states, the original flow environment could be restored which serves as a reference for the virtual treatment target. It was shown that the realistic deployment led to a considerable stabilization of the individual hemodynamics in all cases. Furthermore, a quantification of the stent-induced therapy effect became feasible for the treating physician. The results of the morphological and hemodynamic analyses in this pilot study show that virtual stenting can be used in FIAs to quantify the effect of the planned endovascular treatment.

## 1. Introduction

Fusiform intracranial aneurysms (FIAs) are circumferential dilatations of the cerebral vessels leading to pathological hemodynamics. As a consequence, the vessel wall can be drastically weakened followed by a potential rupture with a subsequent subarachnoid hemorrhage. Compared to saccular intracranial aneurysms, FIAs have a lower prevalence in the Western population, where two large autopsy series indicate a prevalence of only <0.1% (1–3). More recently, this value is estimated to be somewhere between 3 and 13% (4). However, FIAs more often occur at the posterior cerebral circulation, can strongly vary in size (diameter and length), and are more often present in young patients with a higher prevalence in men when compared to saccular IAs which have a 3-fold larger prevalence in women (5).

Due to their rarity, FIAs have not been as well studied as saccular aneurysms including morphological and hemodynamic parameter analysis with respect to rupture risk. For instance, morphological parameter analysis usually requires the presence of an aneurysm neck in order to extract features like maximal height or aspect ratio (6). Furthermore, the individual hemodynamic state is difficult to assess, since a clear distinction between the aneurysmal lumen and the parent vessel can be highly complicated.

Regarding the treatment of FIAs, an increasing number of minimally-invasive techniques exists (7). A multicenter study found that FIAs possess the lowest occlusion rate and were associated with most complications during the interventions (8). Furthermore, sufficient device selections can be complicated and undesired treatment effects such as intimal hyperplasia have been shown to be related to insufficient flow-diverter placement (9, 10).

This pilot study focuses on the morphological and hemodynamic analysis of these rare pathologies by comparing three representative FIAs. For assessing the potentially pathologic hemodynamics leading to the formation of the aneurysms, all FIAs were manually removed for healthy counterparts. Furthermore, realistic treatments using virtual flow-diverter deployments were carried out to quantify the efficacy of each patient-specific treatment scenario. This helps to answer the question of whether endovascular treatment of FIAs restores the healthy pre-pathological hemodynamic state.

## 2. Materials and Methods

This section describes the datasets of our pilot study and explains the subsequent analysis steps.

### 2.1. Patient Data

**Figure 1:**
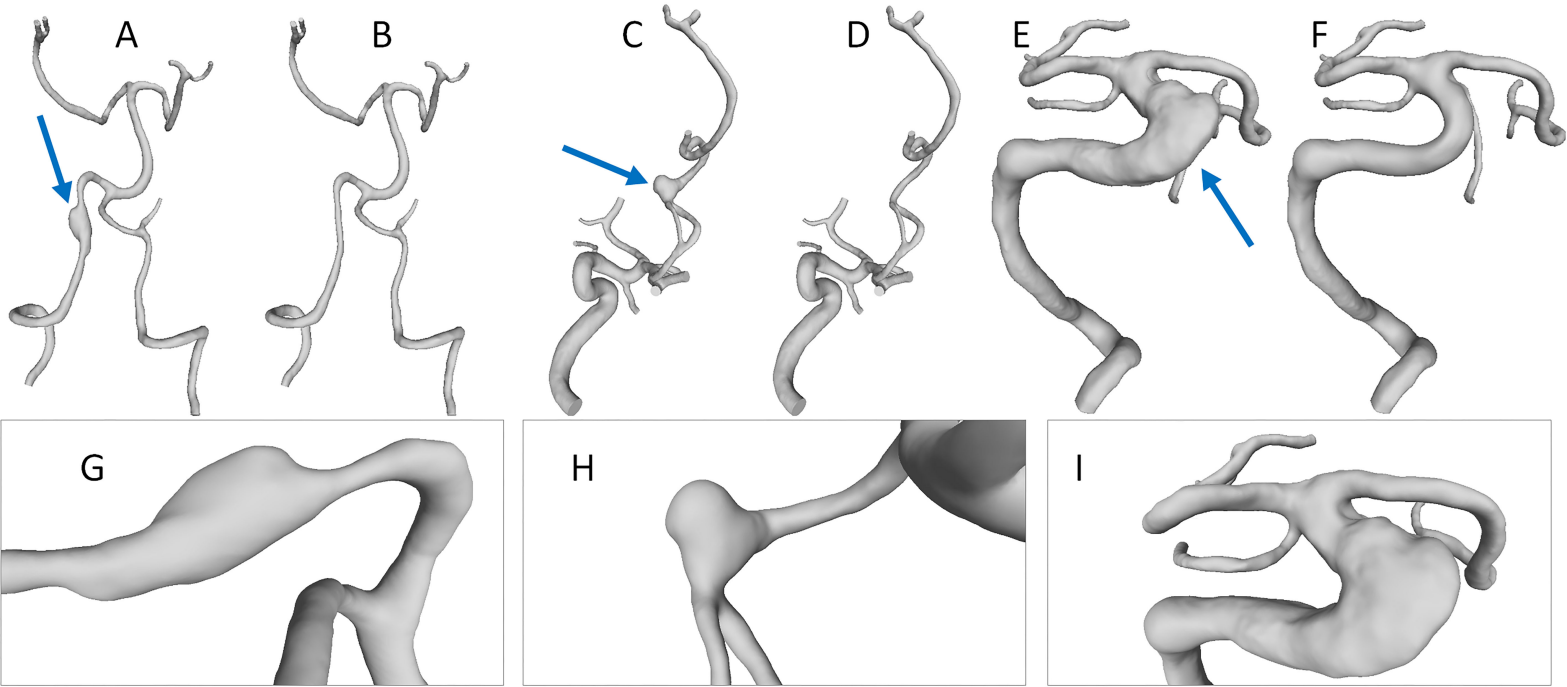
Depiction of the three fusiform aneurysm cases (marked with an arrow) and their corresponding healthy 3D models. Case 1 is depicted in **(A,B)**. Case 2 is depicted in **(C,D)** and Case 3 is depicted in **(E,F)**, respectively. Furthermore, for each fusiform aneurysm case, a detailed presentation is given in **(G–I)**.

Due to the absence of high-quality image data, only three patient-specific fusiform aneurysms were analyzed in this study (refer to Figure 1). The selection was based on Huber's definition, where the lesions present with an arterial dilation larger than 1.5× of the nominal vessel diameter without possessing a neck. Since FIAs are not as common as their saccular counterparts, there was only a limited number available.

For Case 1, a 63-year-old male patient underwent computer tomographic angiography with a resolution of 0.47 × 0.47 × 0.5 mm^3^. The patient suffered from an FIA at the left vertebral artery (Refer to Figure 1). Furthermore, he presented with a subarachnoid hemorrhage which required immediate treatment. Although the stenting was considered successful, the patient suffered from generally poor health and no long-term information was available. For Case 2, a 34-year-old female patient with a FIA at the left middle cerebral artery underwent 3D digital subtraction imaging (3D DSA) with a resolution of 0.28 × 0.28 × 0.28 mm^3^. The patient stopped treatment and did not appear for follow-up visits. Case 3 was acquired as 3D DSA data (0.36 × 0.36 × 0.36 mm^3^) from a 48 years old man with a basilar fusiform aneurysm. A Leo stent (5.5 × 75) was implanted, yielding the desired neurological improvement and aneurysm elimination.

### 2.2. Segmentation and Extraction of the Healthy Counterparts

For each of the patient-specific fusiform data sets, a threshold-based segmentation was applied to yield 3D surface models based on prior work (11). During segmentation, we have been focusing on a large vascular domain, i.e., keeping as much of the peripheral arteries as possible depending on the acquired image data (refer to Figure 1). For subsequent post-processing steps, the vessel centerline was extracted with the Vascular Modeling Toolkit (VMTK) (12). In order to restore the healthy variants of the three FIA models and to compare them with the original shapes, manual Laplacian smoothing was applied locally using the open source 3D creation suite Blender 2.9 (Blender Foundations, Amsterdam, Netherlands). For this purpose, the sculpt mode can be used in Blender 2.9. A circular region of interest is available in which Laplacian smoothing is performed on the corresponding section of the surface. With this tool, the pathological part of the vessel was smoothed and shrunk based on the proximal and distal vessel diameter of the healthy parent vessel. This step was conducted by a single medical engineer.

### 2.3. Fast Virtual Stenting

**Figure 2:**
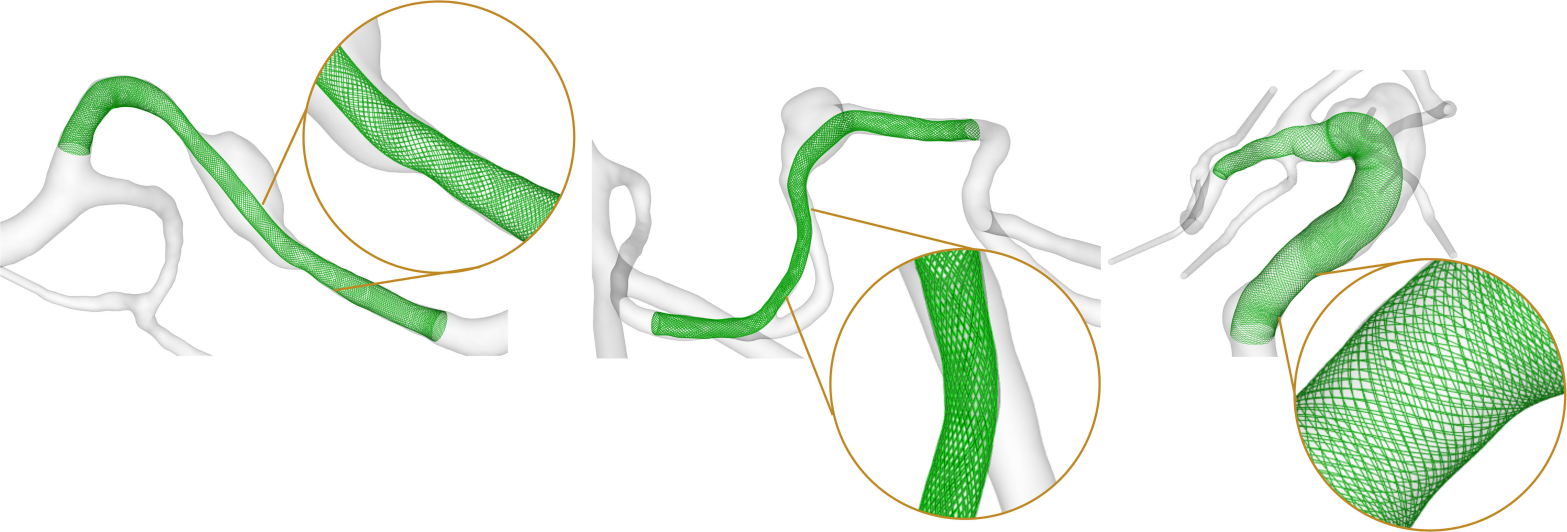
Results of the virtual flow-diverter deployment for Case 1 (left), Case 2 (center), and Case 3 (right). Inlays show the explicit resolution of the braided stent structure allowing for a precise evaluation of the post-interventional hemodynamic state.

To allow for an investigation of the efficacy of endovascular treatment, a fast virtual stenting (FVS) approach was applied to all three FIA cases. A flow-diverting device was virtually deployed using in-house software, which allows for the replication of arbitrary stent properties within minutes (13). The consideration of the discrete stent struts as well as the vessel-induced stent shortening or elongation allows for realistic reproduction of a possible treatment scenario as well as the consideration of flow phenomena occurring in the vicinity of the flow-diverters. The deployment results are displayed in Figure 2, and the corresponding properties are summarized in Table 1. For further details, the interested reader is referred to Berg et al. (14).

**Table 1:** Nominal geometric parameters of the virtual flow-diverter stents for each patient-specific FIA.

**Case**	**Length [mm]**	**Diameter [mm]**	**Number of wires**	**Strut thickness [μm]**
1	20	3	48	42
2	10	2.25	48	42
3	60	7	52	42

### 2.4. Blood Flow Simulation

Image-based blood flow simulations were performed *via* computational fluid dynamics. Spatial discretization of the models resulted in the generation of fine, polyhedral meshes with a base size of 0.15 mm in accordance with recent recommendations in prior study (15). This yielded a total number of elements ranging from 0.8 to 8.9 million, depending on the considered vessel section and configuration. High element numbers are caused by the precise description of individual stent struts.

Due to the absence of patient-specific flow measurements, boundary conditions for in and outflow cross-sections were employed. At each inlet of the considered cases, representative time-dependent flow rates from precise 4D phase-contrast MRI measurement conducted in a healthy volunteer were scaled according with the individual inlets of Cases 1–3 (16). Since the segmented vascular domains contain a high number of outlets, we used an advanced flow-splitting technique for realistic splitting ratios based on the underlying vessel shape (17) instead of the commonly used zero-pressure or generalized approaches based on Murray's. Further assumptions for the time-dependent simulations include the treatment of blood as an incompressible ($\rho =1,055\;\frac{kg}{m^{3}}$), Newtonian (ν = 4 m Pa·s) fluid, and the consideration of laminar flow behavior. For each of the nine simulations, two cardiac cycles were simulated, while the first one was discarded, and the last one was included in the advanced analysis.

### 2.5. Analysis

For the three fusiform aneurysms, qualitative and the quantitative analysis was carried out. Since morphological parameters mostly require a saccular shape, we focused on the vessel cross-section. In order to specifically consider elliptical vessel cross-sections, we utilized previous study to extract the vessel cross-sectional area along the vessel's centerline (17). In this study, rays are cast perpendicular to the vessel's centerline to determine the cross-section of the area.

Next, relevant hemodynamic parameters that were associated with an increased rupture risk in previous studies were extracted. The focus is set on shear-stress and flow-related effects (18–20):

- Time-averaged Wall Shear Stress (*AWSS*)
- Oscillatory Shear Index (*OSI*)
- Relative Residence Time (*RRT*)
- Oscillatory Velocity Index (*OVI*)
- Kinetic Energy (*KE*)

A detailed description of these parameters is provided in the Supplementary Material.

## 3. Results

For each FIA case, the maximum cross-sectional area is evaluated. This is followed by an analysis of the hemodynamic parameters, including a comparison with the manually-created healthy counterparts, and the post-interventional states based on the virtual deployment.

### 3.1. Case 1

For morphological evaluation, we extracted the FIA's cross-sectional areas perpendicular to the vessel's centerline, which are listed in Table 2. The FIA of Case 1, located on the patient's vertebral artery, had a maximum cross-sectional area of approximately 14.53 mm^2^, which is a relative increase in comparison to the parent vessel's of 345%.

**Table 2:** Morphological analysis for each case, where max.

**Case**	**Max. *CSA*_*A*_(mm^**2**^)**	***CSA*_*PV*_(mm^**2**^)**	**RI(%)**
1	14.53	4.21	345
2	7.40	1.24	581
3	70.51	22.61	312

*CAS_A_ denotes the maximum cross-sectional area (perpendicular to the centerline) of the fusiform aneurysm and CSA_PV_ denotes the cross-sectional area (perpendicular to the centerline) of the parent vessel proximal to the aneurysm. RI denotes the relative increase as a percentage, i.e., (max. CSA_A_ / CSA_PV_·100%)*.

**Figure 3:**
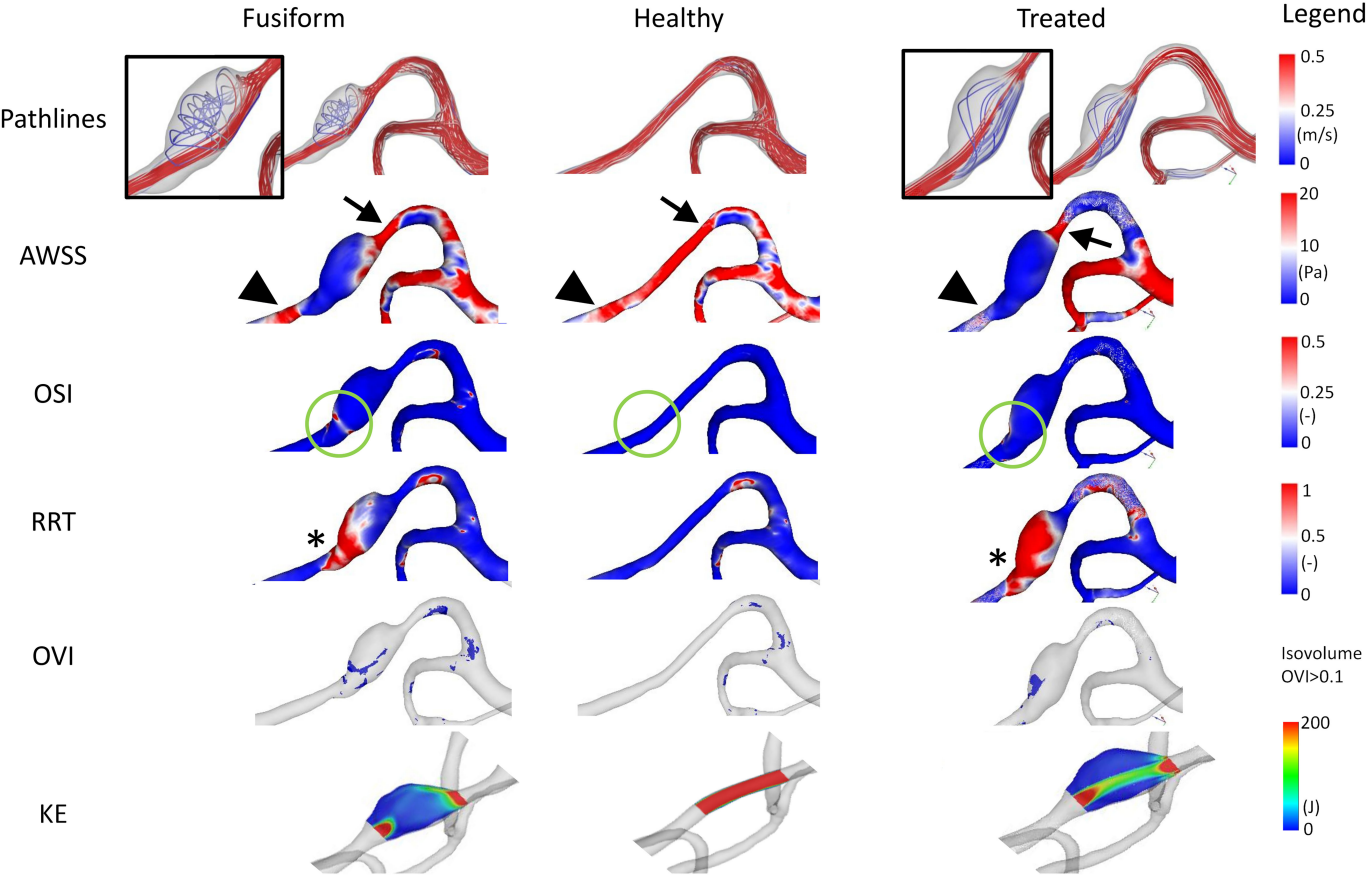
Illustration of the hemodynamic results for Case 1. Fusiform (left) denotes the 3D model of the FIA; Healthy (center) the manually-created healthy counterpart; Treated (right) the 3D model after virtual stenting. The following flow- and shear-related parameters are presented from top to bottom: Pathline visualization, *AWSS*, *OSI*, *RRT*, *OVI*, *KE*. For illustration purposes, view points are not always identical but chosen such that the most interesting features are visualized.

Selected results of the time-dependent blood flow simulations are presented in Figure 3. The blood flow pattern for the FIA shows a small vortex in the aneurysmal lumen indicating a complex flow structure (refer to inlay). When analyzing the healthy model, no vortex or disturbance of the blood flow is perceivable. The simulation of the virtual stenting configuration yields a hemodynamic state, where no vortex remains (refer to inlay). When analyzing the *AWSS*, an area with elevated *AWSS* values is apparent proximal to the aneurysm (refer to arrowheads, Figure 3), which drastically decreases in the aneurysmal lumen. Distal to the aneurysm, an area with higher *AWSS* values remains, independent of the healthy or virtually treated versions (refer to arrows, Figure 3). Regarding *OSI*, the post-interventional configuration yields a reduction of this parameter as well (refer to green circles, Figure 3), which is desired as visible in the healthy version, where no elevated *OSI* is perceived. This is also reflected by the *RRT* analysis. In this study, virtual stenting yields a more homogeneous distribution of blood flow with respect to *RRT* when compared to the untreated dataset (refer to asterix in Figure 3).

Besides the shear-related quantities, the analysis of *OVI* and *KE* enables an assessment of the time-dependent dynamics within the vascular lumen. Specifically, regions of increased oscillatory velocity are present in the FIA Case 1, which vanish in the corresponding healthy vessel section (refer to Figure 3). After virtually deploying a flow-diverting device, these regions can be reduced toward a healthy state. However, a small region of increased flow disturbance remains in the distal part of the aneurysm, which is also detectable by *KE*.

### 3.2. Case 2

The FIA comprises two inlets, a challenge for the treating physician. Although it is smaller than the other cases, it exhibits the largest increase of diameter compared to the parent vessel, recall Table 2. Hence, the maximum cross-sectional area of the FIA is approximately 7.40 mm^2^, which is 581% of the parent vessel's cross-sectional area of 1.24 mm^2^. Compared to Case 1, the FIA presents with a more spherical shape, and due to the relatively small volume, no complex flow patterns develop within the sac.

**Figure 4:**
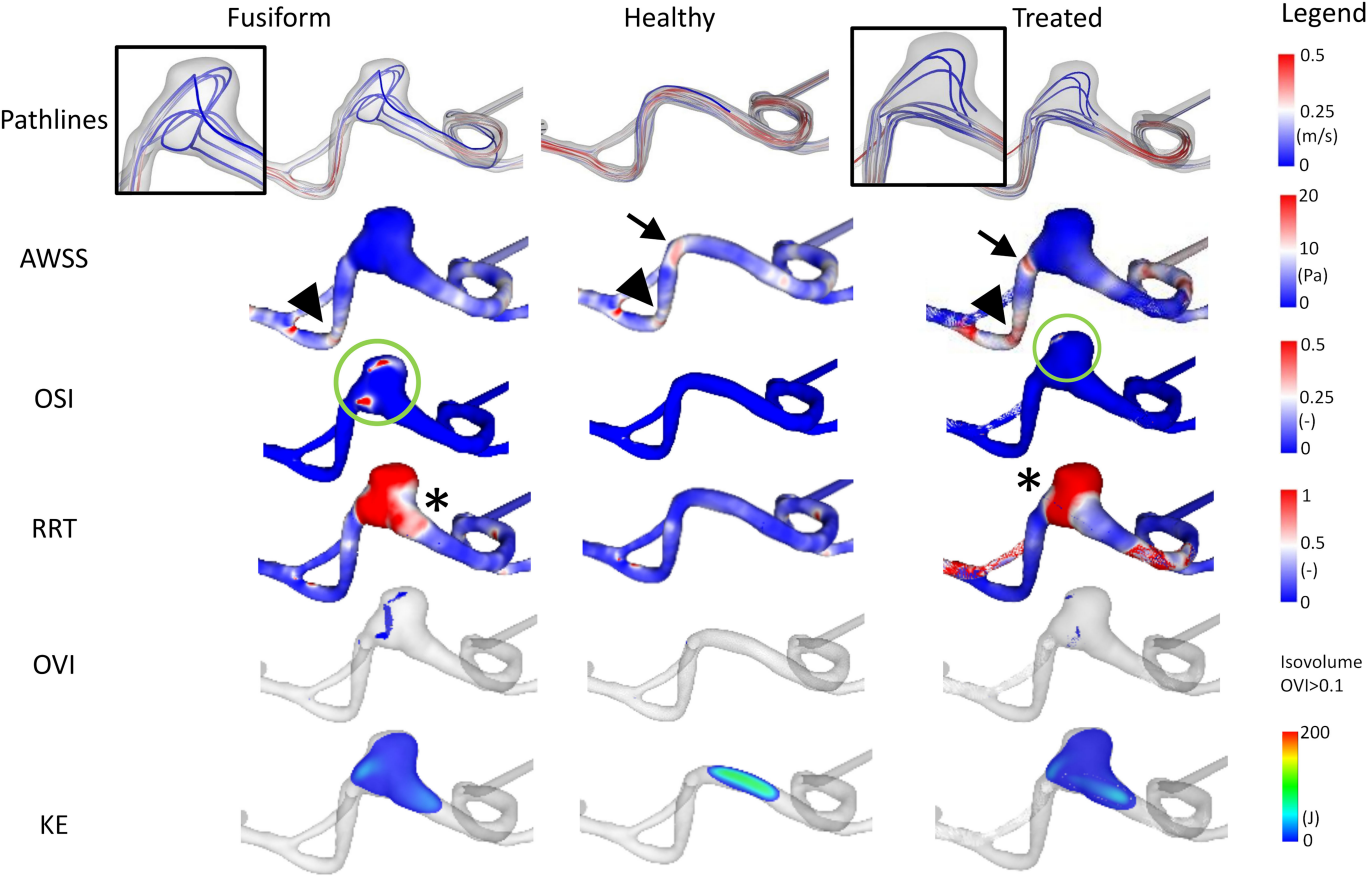
Illustration of the hemodynamic results for Case 2. Fusiform (left) denotes the 3D model of the FIA; Healthy (center) the manually-created healthy counterpart; Treated (right) the virtually stented model. The following flow- and shear-related parameters are presented from top to bottom: Pathline visualization, *AWSS*, *OSI*, *RRT*, *OVI*, *KE*.

However, when analyzing the shear-related parameters, an area of slightly elevated *AWSS* values is apparent in the manually-created healthy version and in the virtually-treated one (refer to arrowheads in Figure 4). In the FIA model, this area has considerably lower *AWSS* values. Furthermore, *OSI* analysis reveals a clear reduction of this hemodynamically relevant quantity when comparing the untreated model with the virtually stented model (see green circles in Figure 4). In contrast, evaluation of *RRT* yields a more heterogeneous distribution, i.e., a smaller area with increased *RRT* values (see asterix in Figure 4).

Regarding the underlying dynamic flow field, high *OVI* values predominantly occur in the fusiform environment and almost vanish after stent deployment (although not completely reduced as noticeable for the healthy case). As observed by the pathlines and due to the distal location within the Circle of Willis, the corresponding kinetic energy appears to be relatively small.

### 3.3. Case 3

Analysis of the cross-sectional area of the FIA yields a maximum area of 70.51 mm^2^ and a cross-sectional area of 22.61 mm^2^ proximal to the aneurysm. Hence, dilation of the basilar artery is visible, with a relative increase of 312%, recall Table 2.

**Figure 5:**
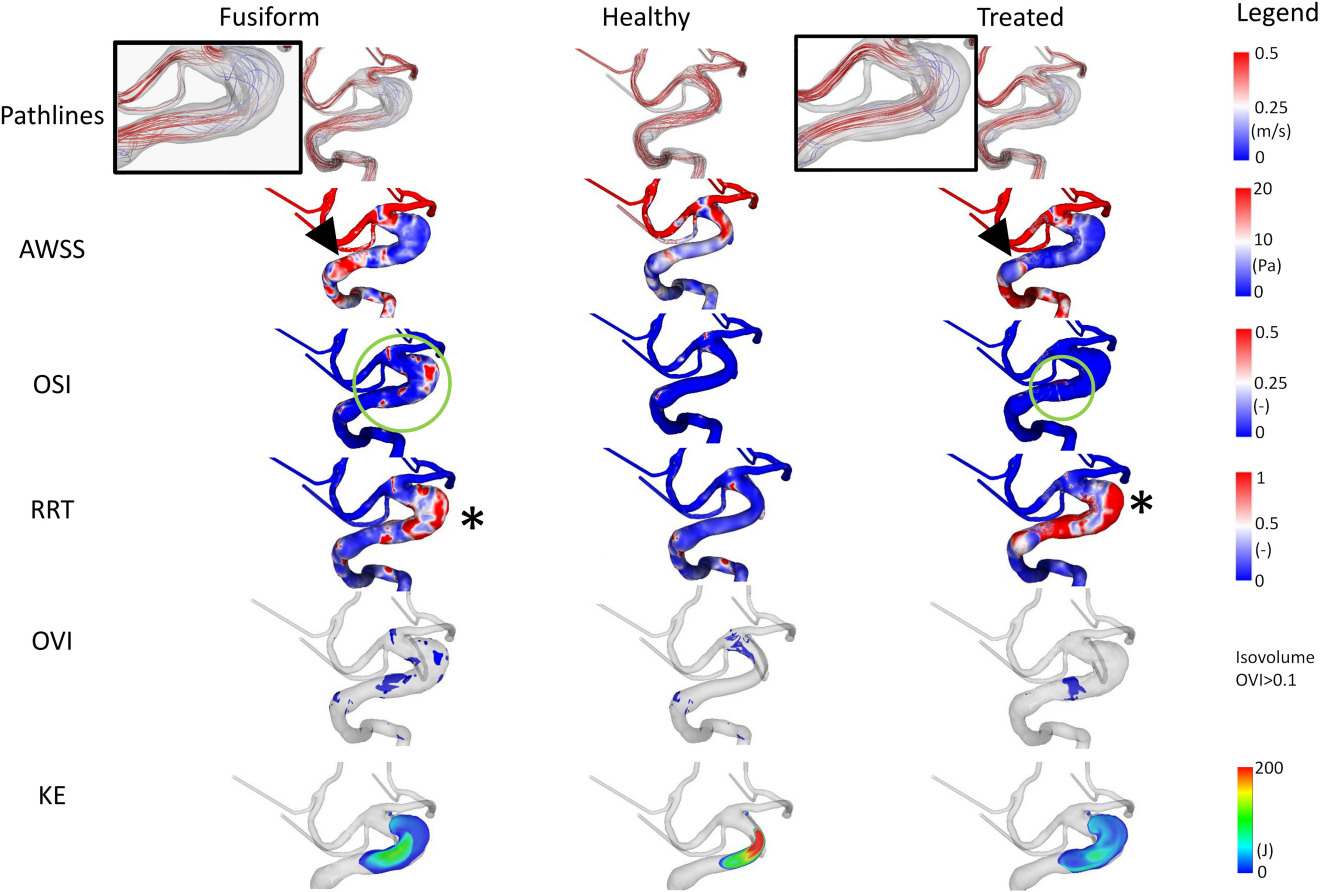
Illustration of the hemodynamic results for Case 3. Fusiform (left) denotes the 3D model of the fusiform intracranial aneurysm; Healthy (center) the manually-created healthy counterpart; Treated (right) the 3D model after virtual stenting. The following flow- and shear-related parameters are presented from top to bottom: Pathline visualization, *AWSS*, *OSI*, *RRT*, *OVI*, *KE*.

The simulated blood flow reveals high velocity values occurring in the curved basilar artery vessel section (refer to Figure 5). With the increasing growth of this region, the absolute velocity decreases, while helical flow structures develop. Regarding the *AWSS* analysis, a hot spot (i.e., an area with increased values) is perceivable proximal to the aneurysm (refer to arrowheads in Figure 5). Furthermore, the wall shear stress pattern occurring in the healthy-vessel section drastically changed due to the formation of the aneurysm. After virtually deploying a flow-diverting device, the low-shear stress distribution remains. However, the blood flow is directed toward the basilar tip.

The analysis of *OSI* reveals the existence of complex and risk-related hemodynamics for the FIA. Large patches with increased values exist which do not appear in the healthy counterpart. Impressively, the virtual treatment strongly reduces the oscillation leading to stable flow structures. For *RRT*, expected tendencies are visible. While almost no increased values appear in the healthy vessel, increased relative residence times can be noted in the fusiform and treated variant.

The flow-related analysis of *OVI* and *KE* confirms the observations of the previous cases. Oscillations are successfully damped by the implantation of a sufficiently-selected flow-diverting device, and the blood is desirably redirected toward the original vessel's course.

## 4. Discussion

Although the research related to cerebrovascular pathologies such as intracranial aneurysms considerably increased within the last two decades, several questions related to initiation, growth, and rupture remain. Since intracranial aneurysms are mostly present in a saccular shape, fusiform cases are most typically observed when formed at the abdominal aorta. However, a small fraction of intracranial aneurysms possesses fusiform shapes as well, and depending on its appearance and localization, the identification of a sufficient (minimally-invasive) treatment strategy can be difficult.

Day et al. (21) report that hemorrhage was most common for very small lesions, whereas the incidence of bleeding is lowered with larger lesions. However, other studies reported a higher risk of hemorrhage for FIAs larger than 10 mm (22, 23). Furthermore, a very recent MRI study by Sabotin et al. (24) demonstrated the presence of increased contrast enhancement in FIAs, which was associated with microhemorrhage and pathophysiological processes.

To further improve the understanding of FIA hemodynamics, the presented pilot study focused on three representative cases due to their rare existence. The localizations are well fitting to literature, where it is stated that FIAs usually occur in the vertebrobasilar system (like Case 1 and Case 3) and that FIAs in the anterior circulation remain rare and occur mostly in the middle cerebral artery (like Case 2) (4). In addition, two of the three cases stem from men, which is also typical for FIAs.

Conducting highly-resolved hemodynamic simulations in three patient-specific FIAs enabled the assessment of locally-occurring flow structures. Specifically, velocity-encoded pathline visualization allows for the qualitative identification of flow phenomena that might progress undesired effects, which repeatedly occur in each cardiac cycle (recall Figures 3–5). Furthermore, the precise calculation of existing *AWSS* patterns demonstrated that all FIAs were subject to abnormally low shear stress values, which can lead to further weakening of the vessel wall and subsequent growth or even rupture (25–27). The recreation of the pre-pathological states revealed that considerably larger *AWSS* values were present in the regions of interest even when compared to other sections of the segmented vasculature. This observation promotes the theory that high shear is related to the initiation of intracranial aneurysms due to triggering a muralcell-mediated destructive remodeling (28).

In addition to the shear stress evaluation, the analysis of *OSI*, which was repeatedly associated with intracranial aneurysm rupture (29, 30), impressively demonstrates the effect of minimally-invasive endovascular treatment. All FIA cases were subjected to abnormally high oscillatory shear values, which were not observed in any healthy counterparts. Due to the virtual deployment of a flow-diverting device, this hemodynamically unstable environment could be restabilized leading to a strong reduction of *OSI*. Although *RRT* was correlated with aneurysm rupture at well (31), its benefit with respect to treatment outcome evaluation remains limited and this trend was not reflected in our study.

When analyzing *OVI*, i.e., a scalar field containing all temporal changes of the velocity vector directions (20, 32, 33), the virtual treatment almost fully recovered the healthy states in all cases demonstrating the efficacy of such devices. Finally, the illustration of the kinetic energy distribution allows for the observation of the desired treatment outcome as well as the identification of undesired flow effects such as concentrated inflow jets.

Regarding minimally-invasive therapy, stenting of FIAs is always challenging since the opening of the flow diverter differs from the behavior observable in saccular cases. Thus, the fast virtual stenting approach allows for testing different stent configurations and provides initial feedback on how strongly the stent is shortened/elongated based on the diameter along the FIA's centerline. For example, the actual stent used in Case 3 was longer than the virtually implanted flow diverter. Although other parameters such as catheter size must be taken into account, virtual planning tools provide valuable information for testing different configurations. Furthermore, undesired treatment effects such as incomplete wall apposition can be evaluated as it can be noticed for Case 1 in the distal part of the FIA (e.g., visualized by the kinetic energy distribution).

Within this study, several limitations exist. First, although our internal database comprises approximately 400 intracranial aneurysms, we were only able to identify three FIAs with sufficient image quality. Hence, only for two of them, post-interventional information was available since one patient stopped treatment. When compared to literature, the expected amount of cases would be even worse, since two large autopsy studies with a combined case number of more than 16,000 reported only 15 fusiform intracranial aneurysms (1, 2). Second, we conducted segmentation of 3D models and virtual removal of the fusiform aneurysms that include manual processing and thus might suffer from user specific variations. Third, the blood flow simulation requires several assumptions, which might not be able to perfectly reflect reality (15). However, extensive validation studies were carried out in advance to demonstrate the accuracy of image-based simulation (34). Finally, the rupture risk prediction of aneurysms is a challenging task, where longitudinal studies would be required to truly assess the individual rupture risk.

## 5. Conclusion

In this pilot study, we analyzed the pathological hemodynamics in three representative fusiform intracranial aneurysms based on a multi-modal simulation approach. By manually restoring the healthy hemodynamic environment for each case, reference flow conditions could be obtained for comparison. Furthermore, minimally-invasive endovascular therapy was performed in two of the patients based on a fast virtual stenting approach confirming the efficacy of flow-diverting devices for this neurovascular pathology.

Although we could demonstrate our findings only for a very limited number of cases due to the rarity of fusiform aneurysms, our results reflect the potential for blood flow analysis and virtual stenting of these pathologies. For future study, the analysis of a considerably larger amount of FIAs is required to confirm our initial finding.

## Data Availability Statement

The raw data supporting the conclusions of this article will be made available by the authors, without undue reservation.

## Author Contributions

PB and SS: conceptualization, formal analysis, resources, supervision, project administration, and funding acquisition. LM, PB, and SS: methodology. JK, JS, and SS: software. JK, LM, and PB: validation. JK, JS, LM, PB, and SS: investigation. OB, YC, and DB: data curation. PB, JS, and SS: writing—original draft preparation and visualization. JS, LM, BP, DB, PB, and SS: writing—review and editing. All the authors have read and agreed to the published version of the manuscript.

## Funding

This study was funded by the German Federal Ministry of Education and Research within the Research Campus STIMULATE (grant no. 13GW0473A) and the German Research Foundation (grant nos. SA 3461/3-1 and BE 6230/6-1).

## Conflict of Interest

DB is a Proctor/Advisor: Phenox, Balt, Acandis, Thromb X, Perflow, Kaneka, Penumbra, outside of the submitted work. The remaining authors declare that the research was conducted in the absence of any commercial or financial relationships that could be construed as a potential conflict of interest.

## Publisher's Note

All claims expressed in this article are solely those of the authors and do not necessarily represent those of their affiliated organizations, or those of the publisher, the editors and the reviewers. Any product that may be evaluated in this article, or claim that may be made by its manufacturer, is not guaranteed or endorsed by the publisher.
