# Supplementary material for: Can Endovascular Treatment of Fusiform Intracranial Aneurysms Restore the Healthy Hemodynamic Environment?–A Virtual Pilot Study
Source: Front Neurol. 2022 Jan 24;12:771694. doi: 10.3389/fneur.2021.771694 (PMC8818669; doi:10.3389/fneur.2021.771694)
Supplement: Supplementary file 1 [file Data_Sheet_1.PDF]

Supplementary Material

In the following, the investigated hemodynamic parameters are described in detail.

- Time-averaged Wall Shear Stress ($AWSS$) describes the tangential shear stress along the luminal vessel wall. To obtain a representative vector field, the time-dependent results of the wall shear stress wss_i were averaged over one cardiac cycle.

$$AWSS = \frac{1}{T}\int_0^T wss_i\, dt \tag{S1}$$

- Oscillatory Shear Index (OSI) is metric to express the change in direction and magnitude of wall shear stress wss_i and therefore indicates unstable local flow structures.

$$OSI = \frac{1}{2}\left\{1 - \frac{\frac{1}{T}|\int_0^T wss_i\, dt|}{\frac{1}{T}\int_0^T |wss_i|\, dt}\right\} \tag{S2}$$

- Relative Residence Time (RRT) provides information about the blood flow distribution at the wall. In this parameter the oscillatory shear index and the averaged wall shear stress are combined. This implements the normalization of the parameters and lowers the strength of the parameter compared to the remaining ones.

$$RRT = [(1 - 2 \cdot OSI) \cdot AWSS]^{-1} \tag{S3}$$

- Oscillatory Velocity Index (OVI) characterizes the fluctuation of the fluid flow within a time period. The parameter is calculated from the velocities over time and implies the impact of the vorticity inside the vessel flow. A low OVI implies the continuous blood flow over a heart cycle. The OVI increases with the changes in fluctuation and therefore vortex formation over time.

$$OVI = \frac{1}{2}\left\{1 - \frac{\frac{1}{T}|\int_0^T V\, dt|}{\frac{1}{T}\int_0^T |V|\, dt}\right\} \tag{S4}$$

- Kinetic Energy (KE) describes the part of the energy, which occurs because of the movement of the flow. The density ρ and squared velocity V is considered for the calculation.

$$E_{kin} = \frac{1}{2}\rho V^2 \tag{S5}$$
